# Amphipathic Substrates Based on Crosslinker-Free Poly(ε-Caprolactone):Poly(2-Hydroxyethyl Methacrylate) Semi-Interpenetrated Networks Promote Serum Protein Adsorption

**DOI:** 10.3390/polym12061256

**Published:** 2020-05-30

**Authors:** Guillermo Vilariño-Feltrer, Alfredo Salgado-Gallegos, Joan de-la-Concepción-Ausina, José Carlos Rodríguez-Hernández, Mohsen Shahrousvand, Ana Vallés-Lluch

**Affiliations:** 1 Centre for Biomaterials and Tissue Engineering, Universitat Politècnica de València, 46022 Valencia, Spain; guivifel@upv.es (G.V.-F.); alfredosgallegos@gmail.com (A.S.-G.); joadela1@etsii.upv.es (J.d.-l.-C.-A.); jorodhe1@ter.upv.es (J.C.R.-H.); 2 Caspian Faculty of Engineering, College of Engineering, University of Tehran, P.O. Box 119-43841 Rezvanshahr, Iran; mohsen.shahrousvand@gmail.com; 3 Biomedical Research Networking Centre in Bioengineering, Biomaterials and Nanomedicine (CIBER-BBN), 46022 Valencia, Spain

**Keywords:** poly(ε-caprolactone), poly(2-hydroxyethyl methacrylate), amphipathic, amphiphilic, crosslinker-free, semi-interpenetrated network, protein adsorption, electrospinning

## Abstract

A simple procedure has been developed to synthesize uncrosslinked soluble poly(hydroxyethyl methacrylate) (PHEMA) gels, ready for use in a subsequent fabrication stage. The presence of 75 wt % methanol (MetOH) or dimethylformamide (DMF) impedes lateral hydroxyl–hydroxyl hydrogen bonds between PHEMA macromers to form during their solution polymerization at 60 °C, up to 24 h. These gels remain soluble when properly stored in closed containers under cold conditions and, when needed, yield by solvent evaporation spontaneous physically-crosslinked PHEMA adapted to the mould used. Moreover, this two-step procedure allows obtaining multicomponent systems where a stable and water-affine PHEMA network would be of interest. In particular, amphiphilic polycaprolactone (PCL):PHEMA semi-interpenetrated (sIPN) substrates have been developed, from quaternary metastable solutions in chloroform (CHCl_3_):MetOH 3:1 wt. and PCL ranging from 50 to 90 wt % in the polymer fraction (thus determining the composition of the solution). The coexistence of these countered molecules, uniformly distributed at the nanoscale, has proven to enhance the number and interactions of serum protein adsorbed from the acellular medium as compared to the homopolymers, the sIPN containing 80 wt % PCL showing an outstanding development. In accordance to the quaternary diagram presented, this protocol can be adapted for the development of polymer substrates, coatings or scaffolds for biomedical applications, not relying upon phase separation, such as the electrospun mats here proposed herein (12 wt % polymer solutions were used for this purpose, with PCL ranging from 50% to 100% in the polymer fraction).

## 1. Introduction

Saying that polymers based on 2-hydroxyethyl methacrylate (HEMA) are the most studied and medically applied hydrogels should stir little controversy, especially amongst biomedical researchers and clinicians. Only 16 years after this family of polymeric materials was proposed for biological use for the first time, the poly(2-hydroxyethyl methacrylate) (or PHEMA, referred to as poly(glycol monomethacrylate) back in 1960 [1]) was being synthesized for at least 13 different biomedical purposes. Those included drug delivery, corneal surgery, soft contact lenses, device coating, or fabrication of a water-swellable spongy prosthesis [2]. The interest on material substrates based on this molecule has ever since increased [3], although current approaches to enhance some properties of biomaterials include the synergistic addition of complementary moieties (namely through copolymerisation [4], polymer blending [5], or graft polymer chemistry [6]).

The most straightforward procedure for combining features from macromolecules of different nature (e.g., lipophilic and hydrophilic) is to blend them under metastable thermodynamic conditions and tighten their spatial distribution by creating new bonds between one or more types of the involved polymer chains. These are known as semi-interpenetrating (sIPN) or interpenetrating networks (IPN), respectively, and are really affordable for production in terms of chemical complexity. Thus, it is no surprise that in the last two decades new sIPN and IPN have been designed to strengthen the biocompatibility [7], the biodegradability [8], the chemical resistance [9] or the elastomeric attributes [7,9] of bare PHEMA hydrogels, or to add antibacterial [10] or thermo-responsive traits [11]. While the different elements comprising an IPN are separated in microscopic pure phases (typically 0.1–10 μm) [12,13], evidences show that strong interactions appear at the interface of the different domains in the presence of water [8,12,13,14]. Such engagements are not covalent bonds [14,15] and may be absent in some blends, though, since they rely strictly on the composition of the IPN [16].

The distribution of functional groups on PHEMA macromolecules is likely responsible for the active forces between phases on these special networks, albeit most authors seem unaware of this when developing such IPN. The vast majority of works in this field outline that an additional multifunctional molecule is required to crosslink the linear chains of PHEMA in order to prevent a later phase separation or the elution of PHEMA segments when swollen in an aqueous medium. That concern is based upon the conviction that (a) small traces of 0.1 wt % of ethylene glycol dimethacrylate (EGDMA, which is chemically akin to HEMA and frequently produced along with it) lead to a covalently crosslinked structure and that (b) several radical initiators produce chain transfer reactions in PHEMA synthesis [17]. However, PHEMA with initial 0.21 wt % of EGDMA in the reagent mixture was found to be soluble in HEMA and HEMA/H_2_O solutions even at high degrees of conversion (MW > 10^5^ Da) [18]. Other researchers used PHEMA without crosslinker and described its long-term swelling (yet not dilution) in water [19,20,21]. In addition, it is possible to prevent chain transfer reactions in radical PHEMA synthesis by adding a non-competitor solvent (typically over 60 wt %) such as aliphatic alcohols [22,23] or H_2_O [24]. According to Ratner [25], there must exist a secondary crosslink based on hydrogen bonds from the terminal hydroxyl groups of HEMA, which subsequently should be highly responsive to polymer tacticity [22]. A spontaneous physical bonding between PHEMA segments can easily explain both its insolubility in water and the stability of multicomponent systems on its sIPN.

Organosilicon compounds, polyurethanes and polyesters like poly-ε-caprolactone (PCL) are typical hydrophobic candidates to be combined with PHEMA, mostly due to the preeminent role of these polymer groups in support or the active regeneration of certain damaged tissues. However, most of the above-cited studies regarding PHEMA sIPN consider the hydrophobic counterpart as fillers for PHEMA hydrogels, overlooking the possibility of using HEMA for the improvement of -for instance- the already satisfactory PCL-cell interaction. To the best of our knowledge, a single publication has surveyed the effect of creating an IPN in which PHEMA is not the richer component, based on an intricate, sequential process [26]. Meanwhile, a recent paper published by one of the present authors reveals the key impact of PHEMA chains (intertwined with PCL diol-based polyurethanes in a sIPN) on the osteogenic differentiation of stem cells, even in the presence of minimal amounts of PHEMA [27]. 

Hence, this original research aims to develop a novel, and yet simple, substrate based on [net-poly (2-hydroxyethyl methacrylate)]-sIPN-poly-ε-caprolactone without the need of a crosslinking agent. Subsequently, consideration has been given to how sIPN heterogeneous microdomains influence the selective adsorption of proteins from foetal serum. The key challenge of the described sIPN is to delay the spontaneous physical crosslink of the PHEMA segments during their synthesis until PCL chains are homogeneously distributed throughout the mass of the sIPN. The described method to deploy a water-insoluble sIPN is conceived to be as simple as possible in order to effectively impact on the development of implantable degradable devices for bioengineering. 

## 2. Materials and Methods 

*Materials:* 2-hydroxyethyl methacrylate (HEMA), benzoyl peroxide (BPO, 75%), fetal bovine serum (FBS), Dulbecco’s phosphate buffer saline (DPBS), Coomassie Blue R-250 and acetic acid were purchased from Merck (Darmstadt, Germany). Dimethylformamide (DMF), Chloroform (CHCl_3_) and methanol (MetOH) were purchased from Scharlab (Barcelona, Spain). Poly-ε-caprolactone (PCL, 43–50 kDa) was purchased from Polysciences (Hirschberg Bergstrasse, Germany). All used without further purification, unless otherwise specified. Buffers and solutions prepared for protein handling were destaining solution (30% *v/v* methanol and 10% *v/v* acetic acid), loading buffer (10% *w/v* sodium dodecyl sulphate, 20% *v/v* glycerol, 0.05% *w/v* bromophenol blue, 2-mercaptoethanol 10 mM and Tris-HCl 0.2 M), running buffer (0.1% sodium dodecyl sulphate, glycine 200 mM and Tris-HCl 25 mM), separating gel solution (0.1% *w/v* sodium dodecyl sulphate, 0.1% *w/v* ammonium persulfate, 0.1% *v/v* TEMED, TRIS base 0.4 M and differing amounts of acrylamide/bis-acrylamide 30/0.8% *w/v*) and stacking gel solution (same as separating gel solution for a final acrylamide concentration of 4% *w/v*). Reagents purchased from Merck, except from aforementioned solvents and sodium dodecyl sulphate (Thermo Fisher Scientific, Waltham, MA, USA). 

*Synthesis of Uncrosslinked PHEMA:* The control of spontaneous physical crosslinking of PHEMA chains during radical polymerisation was first assessed by adding affine solvents to the reagent mixture. MetOH or DMF were used for this purpose, and BPO was selected as the thermal initiator. A crosslinking agent was intentionally avoided while -as stated in the introduction- traces of ethylene glycol dimethacrylate are typically found in commercial HEMA (purity of HEMA in the context of this study ≥ 97.9%, determined by GC). Reagent solutions were prepared at 0, 30, 40, 50, 60, 70, 80, 90 wt % of either MetOH or DMF. While HEMA concentration was dependent on the amount of solvent (from 99.5 to 9.5 wt %), BPO was kept at 0.5 wt % in every solution, in order to ensure a homogeneous initiation of the monomer molecules. Zip bags (40 × 60 mm) were confined between two 15 × 15 cm glass plates with a 1 mm-PTFE spacer. The reagent solutions were poured in the zip bags, to be sealed during the reaction that took place in an oven at 60 °C for 24 h. The temperature was enough to ensure a moderate decomposition rate for BPO in solvents [28], but still below MetOH boiling point (T_b_ = 64.7 °C). Once a working concentration of solvents was estimated, new reagent solutions were prepared at 75 wt % of MetOH or DMF and placed in new moulds with zip bags. In this case, the mixtures were set to react likewise at 60 °C for 0, 4, 8, 12, 16, 20, 24, and 28 h. To evaluate the formation of a physically crosslinked network, samples that were completely solid at the end of the reaction were placed in a vial with excess solvent and left overnight. Suitable synthesis conditions should form solid, soluble polymers with jelly consistency-soluble gels hereinafter, to avoid ambiguity with insoluble, crosslinked analogs—that dissolve in excess of solvent, as an evidence of a non-crosslinked polymeric solution being created.

*Assessment of the Network Irreversibility:* The soluble gels (75 wt % MetOH or DMF for 24 h at 60 °C) were air-dried to remove the solvent, thus promoting physical bonding between macromolecules that solvent hindered during polymerization. Then, PHEMA xerogels underwent a controlled swelling with an amount of solvent equivalent to the lost weight. The rheological properties of soluble gels and resulting substances after swelling were measured with a Discovery HR-2 rheometer (TA Instruments, Hüllhorst, Germany) with 25 mm-parallel plates (gap 1 mm) at 25 °C, and angular frequency of 10 rad/s. 

**Scheme 1:**
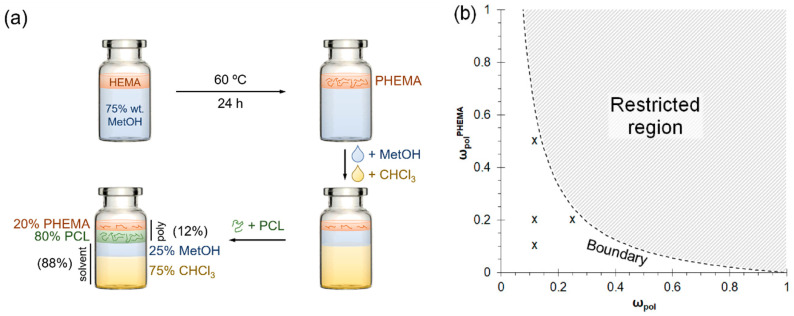
Preparation of PCL- poly(hydroxyethyl methacrylate) (PHEMA) quaternary solutions. (**a**) Example of steps for the preparation of a quaternary 12 wt % polymer blend solution with 80 wt % PCL in the polymer fraction; (**b**) Mass fraction of PHEMA in the polymer fraction, *ω_pol_^PHEMA^*, versus mass fraction of polymer mixture (PCL + PHEMA) in the solution, *ω_pol_*. The dashed line depicts the physical limitation of the fabrication technique. Quaternary solutions used throughout the manuscript are represented with **x’s**.

*PCL-PHEMA Qaternary Solutions:* First attempts in blending PCL with PHEMA 25 wt % soluble gels were unsuccessful regardless of the addition of more solvent, leading irremediably to a two-phase separation of a gelatinous mass. Only MetOH prevented gelation above 35 °C, but only for total amounts of solvent >95 wt % Hence, chloroform (CHCl_3_)—a good solvent for PCL—was included in the formulations to homogenize the blend prior to removing the solvents. The ratio of CHCl_3_:MetOH in the fraction of solvents was set at 3:1 wt. because sIPN rich in PCL were pursued. Nevertheless, such solvent mixture enabled to obtain homogeneous, clear solutions within the physical boundaries of the preparation of the quaternary solutions. For the sake of clarity, Scheme 1 illustrates an example of a 12 wt % polymer blend solution with 80 wt % of PCL in the polymer fraction. All quaternary formulations were mixed with a magnetic stirrer in a sealed ISO flask at 40 °C for 2 h once the PCL was added, until the solution was clear and homogeneous. They were kept refrigerated (5 °C) until further use.

*PCL:PHEMA sIPN Substrates Fabrication:* The metastability of the quaternary solutions prevented using techniques such as solvent casting or phase inversion to achieve thin, flat substrates. Instead, a WS-650-23B spin coater (Laurell Technologies Co., North Wales, PA, USA) was used to obtain solid samples with 50, 80 and 90% PCL in the polymer fraction, along with pure PCL and PHEMA films. Succinctly, 120 µL of quaternary solutions of 12 wt % of polymer blend were dripped on 12 mm-diameter glass coverslips and spin coated at 3000 rpm (a = 1500 rpm^2^) for at least 30 s. While concentrations between 8 and 25 wt % of polymer blend produced satisfactory, macroscopically-uniform substrates, 12 wt % solutions preheated at 35–45 °C were proven more reproducible. Samples of pure PCL and PHEMA were also produced from 12 wt % binary solutions with CHCl_3_ and MetOH, respectively, to ascertain whether the mixture of solvents may cause changes in the thermophysical properties of the polymer substrates. Specimens were named APsIPNx, where x represents the mass fraction of PCL in the solid substrates (hence, pure PHEMA is APsIPN0). To verify the deployment of a sIPN based on physical crosslink even at low moieties of hydroxyl groups from PHEMA, the weight of the samples was studied before and after soaking in saline (72 h) and freeze-drying. Furthermore, substrates were observed under a Carl Zeiss ULTRA-55 FE–SEM (Jena, Germany; Pt sputter coating; EHT = 1 kV), scanned with a PerkinElmer DSC 8000 calorimeter (Waltham, USA) at 20 °C/min, and their contact angle with water was measured (Dataphysics OCA 25) in dry conditions and after 1 h of swelling in water at room temperature (N = 4 each). 

*Protein Adsorption on Amphipathic Substrates*. Considering that cell-substrate interactions are intrinsically mediated through proteins that are selectively adhered on the surface, a serum protein adsorption test was performed. Firstly, FBS was diluted at 10% *v/v*. in sterile DPBS. Then, 200 µL of 10% FBS were added to spin coated substrates with 0 (pure PHEMA), 50%, 80%, 90%, and 100% PCL. Glass coverslips were used as controls, treated with either 10% FBS or DPBS. Samples were incubated for 30 min at 37 °C in a moisture chamber. Following that, 10% FBS was removed and the substrates were rinsed three times with fresh DPBS to induce the desorption of reversibly-adhered proteins. For every condition, a batch of N = 4 replicates was used to check measurements via a Micro BCA Protein Assay Kit (Thermo Fisher Scientific) adapted to flat substrates. Instead of collecting the adsorbed proteins, 100 µL of fresh DPBS were placed on every sample. Next, the Micro BCA working reagent was prepared according to the protocol and 100 µL were added and thoroughly mixed, followed by 2 h of incubation at 37 °C in a moisture chamber. Then, 100 µL were later transferred to a 96-well culture plate and the absorbance of each well was measured at 562 nm on a VICTOR^3^ plate reader (PerkinElmer). Substrates were also analysed with a Bruker Multimode 8 atomic force microscope (AFM, Breika, MA, USA) before and after incubation. In parallel, serum proteins were denaturalized and detached from substrates with 50 µL of loading buffer after incubation and rinsing. Also, the supernatant from the incubation with 10% FBS was stored and diluted 100-fold with loading buffer. An SDS–PAGE was performed with both types of samples (collected proteins and supernatant serum), with independent 6 and 15% *v/w* acrylamide gels (80 V) until significant bands from the standard marker were resolved. Protein signature was then revealed with 0.25% Coomassie Blue R-250 in destaining solution for 2h and later cleared with fresh destaining solution. Analysis of protein signature on the gels was performed with GelAnalyzer software (Lazar, Istvan Jr and Lazar, Istvan Sr).

*Proof of Concept for Tissue Engineering Applications*. Considering the potential application of amphiphilic sIPN for biomedical purposes, some techniques for tissue engineering were considered for a trial. Given the considerable viscosity of quaternary solutions and considering that MetOH and CHCl_3_ have similar boiling temperatures and vapour pressures at room temperature, the samples were deemed adequate for electrospinning. First attempts using home-made equipment (a syringe pump, a voltage source and a collector) proved viscosity of 12 wt % polymer solutions with 20 wt % PHEMA in the polymer fraction to be too low and, thus, a concentration closer to the boundary (see Scheme 1) was selected. A 25 wt % polymer solution with 20 wt % PHEMA was electrospun at 20 kV with a needle-to-collector distance of 15 cm, with controlled temperature and humidity. Electrospun meshes were air-dried and prepared for FE–SEM imaging. In addition, rheological properties of the 12 wt % solution were evaluated for different temperatures and shear strain, in order to determine the optimal range for production.

## 3. Results

### 3.1. Production of Deployable Physically-Crosslinked PHEMA

Table 1 and Table 2 show the appearance and solubility of synthesized PHEMA samples diluted at different weight ratios and for different reaction times, respectively. Solid samples were considered as elastomers (E) if they preserved the shape and thickness of the mould, and gels (G) when the resulting sheets were soft and clearly swelled in solvent. Moreover, the solubility test revealed when physical crosslinks impeded dilution (IS) and when PHEMA macromolecules were able to migrate freely (S), in excess of solvent.

**Figure A1:**
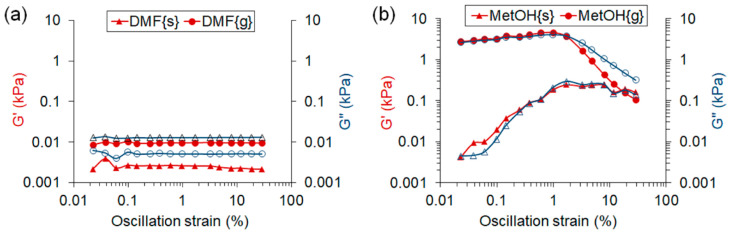
**Rheological properties of PHEMA solutions and gels.** Storage (G’, red) and loss modulus (G”, blue) of PHEMA solutions {s} and “re-swelled” gels {g} in DMF (**a**) or MetOH (**b**).

Apparently, hydroxyl-hydroxyl hydrogen bonds are formed during chain growth of PHEMA oligomers, even when plasticising molecules are present. Only over 60 wt % of polar solvents can this effect can be sterically restricted, in correspondence with the claim made by Gregonis, Russell and Andrade [22]. The phenomenon seems to be independent of the solvent, provided it is considered a “good solvent” for HEMA and PHEMA. Moreover, the spontaneous physical crosslink arises even under high concentrations of MetOH or DMF if the reaction time exceeds for more than 24 h (Table 2). If solutions are cooled down to room temperature under soluble gel (SG) reaction conditions, they remain soluble for days. When stored at 5 °C, the solubility is ensured for several months, even though the polymer network will assemble in this state easily by removing part of the solvent or drying it entirely. Indeed, an analysis of rheological properties of the SG with 75% solvent reacted at 60 °C for 24 h (Figure A1) showed that both MetOH and DMF allowed the formation of a PHEMA gel (G’ > G”) from a viscous liquid solution (G” > G’) by drying, even if the lost solvent is restored.

However, the two types of PHEMA gel performed very differently at low strain, showing MetOH-based crosslinked networks with a complex shear modulus that was at least two orders of magnitude higher (G* ≈ 5 kPa) than those DMF-based networks (G* = 0.01 kPa). Shear-thinning over 2% strain (Figure A1b) also proved the physical nature of crosslinks, reversible under rigorous environmental conditions.

Hence, PHEMA polymerized with 75 wt % MetOH at 60 °C for 24 h was chosen as the hydrophilic, gellable moiety for an interpenetrated network, in order to maximize the molecular weight of the macromolecules without compromising the initial solubility of the gel. 

### 3.2. Characterisation of APsIPN Substrates

#### 3.2.1. Microscopic Imaging and Thermophysical Assessment

The aforementioned solubility of PHEMA was crucial for blending it to its hydrophobic counterpart, the PCL. Conventional procedures for sIPN fabrication—such as network swelling and percolation or in situ polymerisation of monomer in a polymer solution—were restricted due to the different hydrophilicity of functional groups in PCL and HEMA. This led to the incorporation of a second solvent into the polymer mixture.

**Figure 1:**
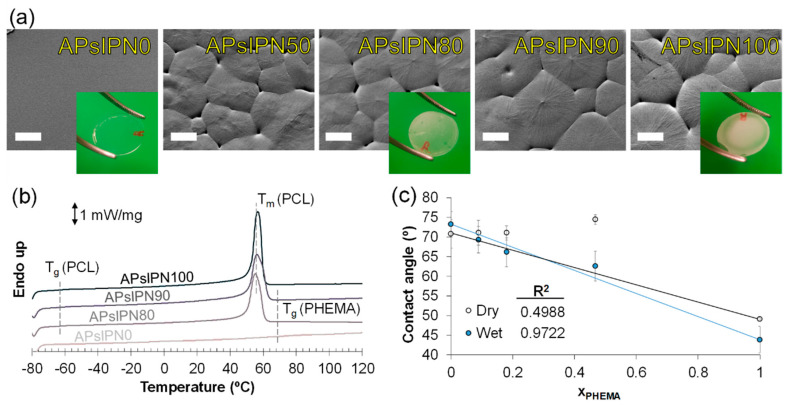
Topography and properties of APsIPN substrates. (**a**) SEM micrographs of amphiphilic semi-interpenetrated (sIPN) substrates with different PCL mass fractions. Details show pictures of selected substrates on coverslips. Scale bar: 20 μm; (**b**) Calorimetry of APsIPN substrates. Melting (T_m_) and glass transition temperatures (T_g_) of pure homopolymers are outlined; (**c**) Contact angle of substrates with different PHEMA moieties in dry (void) and swollen (blue) conditions. Notice that the x-axis represents molar fraction (x), instead of mass fraction (ω).

Even though a quaternary solution involves limitations on its use because of the risk of instability between pairs of components, flat, pale-white, homogeneous substrates were obtained by spin coating APsIPN solutions (Figure 1). Microscopically, the contour and growth front of spherulites 20–40 μm in size were clearly traced (Figure 1a) on substrates with PCL. The intensification of lamella grooves on spherulites along with the higher peak area at 56 °C (Figure 1b) with increasing fractions of PCL proved roughness and micrometric discontinuances to be an exclusive contribution of polycaprolactone crystallisation. Besides, the presence of PHEMA does not seem to intervene in the formation of PCL spherulites. Assuming a ΔH_f_ value of 135.26 J/g for pure crystals (X_c_ = 100%) [29], all APsIPN100 (X_c_ = 43.98%), APsIPN90 (X_c_ = 42.32%), and APsIPN80 (X_c_ = 43.33%) substrates presented virtually the same crystallinity when normalized to the PCL mass fraction.

#### 3.2.2. Behaviour in Aqueous Solutions

Apart from being insoluble in MetOH, APsIPN0 remained stable in water, once deployed (i.e., when the solvent fraction is reduced beyond the boundary in Scheme 1b or removed straight). Actually, gravimetric variations (<0.5% original mass) were not measurable with a precision scale for any sIPN after 72 h of immersion in distilled water, thus displaying a steady persistence in aqueous solutions. 

Figure 1c shows the contact angle of distilled water drops on APsIPN substrates and the predicted values (solid lines) for different molar proportions of PHEMA. While the contact angle of dry substrates seemed independent to the PCL-to-PHEMA molar ratio (R^2^ = 49.88%), the trend changed dramatically when the surface of substrates was exposed to water (R^2^ = 97.22%). This suggests that, although PCL and PHEMA showed some degree of engagement, interfacial functional groups retained a significant mobility. The reorientation of methyl and 2-hydroxyethyl methanoate side moieties around the PHEMA backbone—which has been known for a long time [30]—causes the emergence of hydrophilic portions in the presence of surrounding water, and is typically associated to free (i.e., non-bonded) OH-terminal branches [31]. Accordingly, while APsIPN containing PCL appear to be poorly wettable in air, hydroxyl groups spring up from them when immersed in water. In addition, the proportion of PHEMA repeating units in the initial formulation corresponds to their availability on the surface, a determining factor in protein attachment on the substrates, which in turn has a key role in cell-material adhesion.

### 3.3. Biomedical Approaches of APsIPN

#### 3.3.1. Serum Protein Adsorption on Amphiphilic Substrates

**Figure 2:**
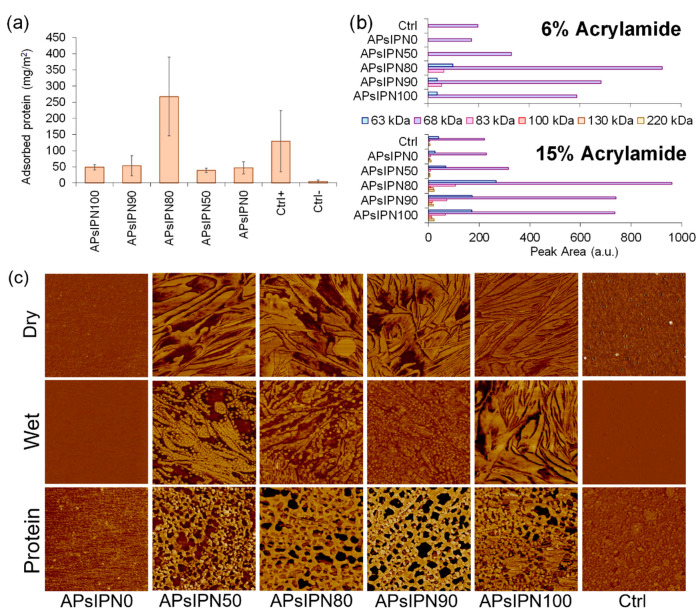
Serum protein adsorption on APsIPN substrates. (**a**) Adsorbed serum protein per area, as measured from the adapted Micro BCA assay; (**b**) Greyscale band quantification of stained SDS–PAGE acrylamide gels (6 and 15 wt %) for detached proteins. Significant bands are indicated as their corresponding molecular mass; (**c**) Phase-contrast atomic force microscope (AFM) images of the array of APsIPN substrates and control (glass coverslip) in dry conditions (top), after a 30-min immersion in water (middle) and after incubation with 10% fetal bovine serum (FBS) (bottom). All samples were sprayed with distilled water and dried with argon prior to observation. Images are 1 μm side squares, normalized to (−30°, 30°) phase range.

**Figure A2:**
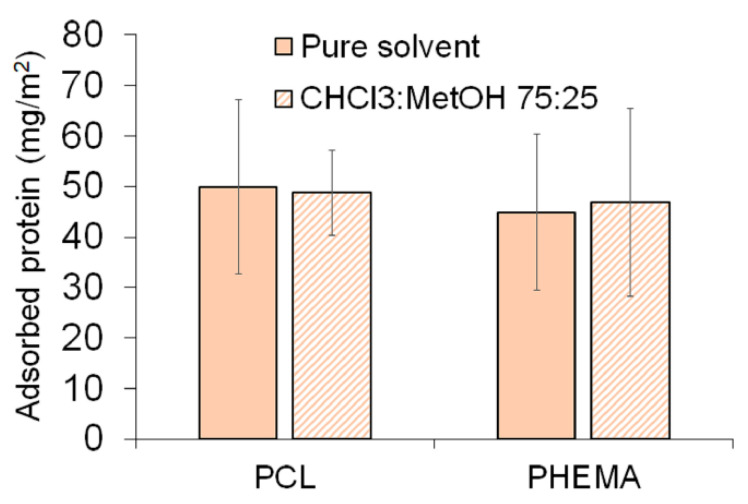
**Effect of solvent over protein adsorption on homopolymer substrates.** Comparison of serum protein adsorbed on substrates based in pure homopolymers (PCL = APsIPN100; PHEMA = APsIPN0). Pure solvents for PCL and PHEMA are CHCl_3_ and MetOH, respectively.

**Figure A3:**
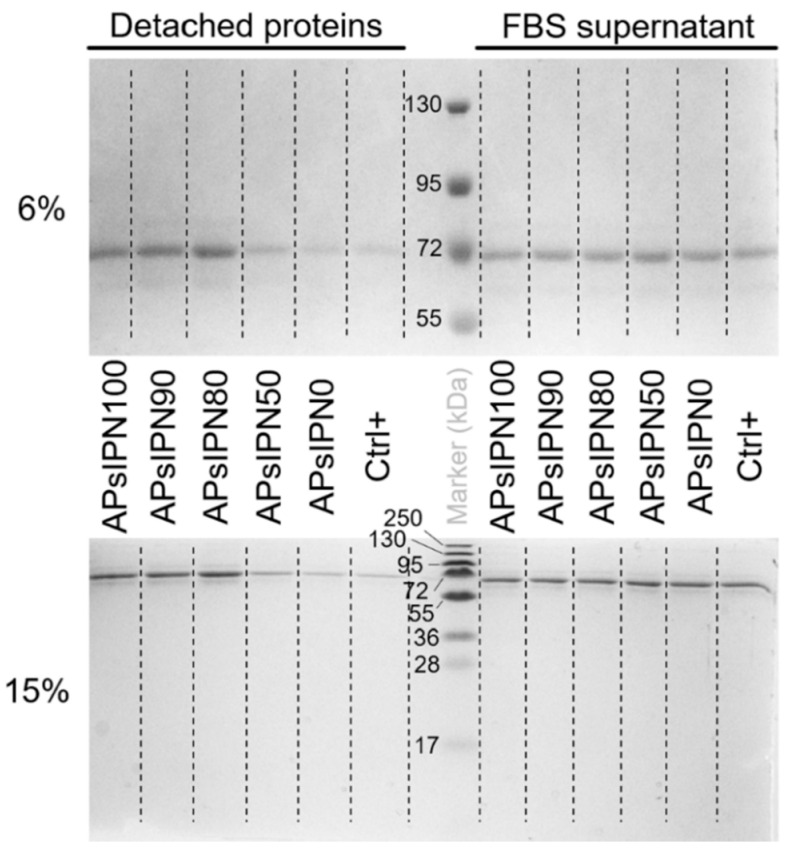
**Protein electrophoresis.** Coomassie blue-stained gels at 6% (top) and 15% (bottom) acrylamide after SDS–PAGE of serum proteins, either detached from the substrates with loading buffer or diluted 1:100 *v/v* from the FBS incubation supernatant.

According to quantifications in Figure 2a,b, total adsorbed protein from bovine serum on APsIPN substrates is dependent on the amount of PHEMA and PCL in the sIPN. Indeed, the coexistence of both types of macromolecules led to an enhancement in the number and stability of interactions between proteins and the polymer, as opposed to bare homopolymers. It is worth noting that pure PCL and PHEMA showed the same behaviour when coated with 10% FBS, regardless of the solvent used for spin coating the samples (Figure A2). There is a noticeable discrepancy among results for substrates rich in PCL (namely APsIPN90 and APsIPN100), though. Such discrepancy is soundly attributable to the modification used for the Micro BCA, since all proteins were chemically detached from the substrate for the test in Figure 2b, yet other alternative explanations are not discarded. An interesting observation was reported regarding protein adsorption results: APsIPN80 (molar ratio x_PCL_ = 0.82) showed a five-fold increase with respect to PHEMA, regardless the analysis assay studied, and thus performing better than an equimolar proportion. The SDS–PAGE was unable to detect differences between the affinity of typical FBS proteins to the substrates, neither for the adsorbed proteins nor for the incubation supernatant (Figure A3). In all cases, the most relevant blot appeared at 68 kDa, and the rest of bands were proportional to the total amount of protein. This signal is typically identified with serum albumin, the most abundant protein present in FBS along with the immunoglobulin superfamily.

Figure 2c displays phase-contrast AFM micrographs of all substrates under study, including the glass coverslip used as control of an adherent substrate. Samples were analysed dried, swollen in water and after being incubated with 10% FBS. While—predictably—pure homopolymers and the glass coverslip remained superficially unaltered when exposed to water molecules, sIPN showed remarkable differences. In contact with air, the images revealed wide sectors with parallel seams, similar to the structure found in APsIPN100. Meanwhile, the “wet” substrates presented numerous rounded, nanometric lumps all over the region, probably due to hydroxyl groups from PHEMA that were previously concealed, what has been explained in the previous section. The typical size of those lumps was 10–20 nm for APsIPN50 and APsIPN90, and 20–45 nm for APsIPN80. This proved the uniformity of all sIPN substrates, since they are clearly below the values of nanophase separation regions often reported by other authors [12,13]. Furthermore, APsIPN80 substrates in contact with water preserved a phase distribution somewhere in between pure PCL and PHEMA, where APsIPN50 and APsIPN90 were closer to PHEMA when hydrated. This might be the reason for APsIPN80 presenting a regular protein lattice while preserving nanophase separation underneath. APsIPN50, APsIPN100, and especially APsIPN90 also facilitate somewhat a protein pattern, moderately and at the expense of ostensibly flattening the seamed surface. By contrast, proteins adsorbed on APsIPN0 and the glass coverslip arranged globularly on clusters of 20–50 nm.

#### 3.3.2. Electrospun Meshes Based on APsIPN

The aforementioned metastability of the quaternary solutions represents a potential hindrance for using certain fabrication procedures, specifically those allowing phase separation during solvent removal. However, several techniques for producing polymer substrates, coatings or scaffolds for biomedical applications bypass this limitation. Such is the case of electrospinning, a method for obtaining meshes of nanometric fibres by propelling a jet of polymer solutions from a needle with the help of an intense electric field.

**Figure 3:**
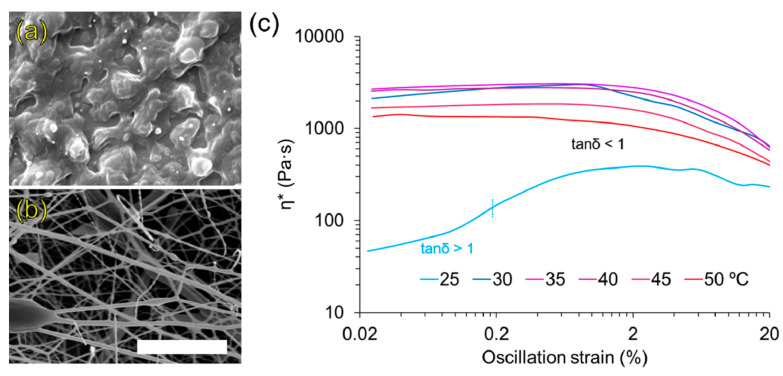
Validation of APsIPN solutions spinnability. SEM micrographs of resulting membranes after electrospinning (**a**) 12 wt % or (**b**) 25 wt % solutions of APsIPN80 in CHCl_3_:MetOH 75:25 (3:1 ratio) at 25 °C. Scale bar: 10 μm; (**c**) Complex viscosity measurements of APsIPN80 12 wt % solutions in CHCl_3_:MetOH 3:1 at different temperatures for a wide range of oscillation strains. Samples revealed to be in gel phase (tan δ < 1), except 12 wt % solution at 25 °C for oscillation strain < 0.2% (as per data in Figure A4).

Although optimal conditions for spin coating APsIPN solutions (i.e., 12 wt % polymer with 20 wt % PHEMA in the polymer fraction) did not lead to electrospun meshes (Figure 3a), select, minor changes in solution properties enable the formation of layers of independent fibres. For instance, concentrating the solution near the boundary in Scheme 1b involves a meaningful increase in its viscosity, resulting in loose, spinnable fibres in the range 500–1000 nm, as shown in Figure 3b. Other sources for improving the potential of APsIPN solutions for electrospinning include increasing oscillation strain/shear stress or the temperature of the fluid, since they show shear-thinning (<30 °C) or shear-thickening (≥30 °C) thermo-responsive behaviour (Figure 3c). Indeed, optimal viscosity and damping factor (tanδ) conditions for electrospinning polyacrylates and PCL have already been reported [32,33]. While these factors influence the result positively, other variations like modifying the CHCl_3_:MetOH ratio might compromise the stability of either the mixture or the Taylor cone [34].

## 4. Discussion

Although applications of PHEMA hydrogels typically involve a covalent crosslink, a simple method for solution-polymerizing PHEMA has been obtained and described here in the absence of any crosslinking agent or catalyst. A wide and thorough set of experiments have allowed finding a narrow range of conditions—monomer concentrations and reaction timespans—in which the arising macromolecules can be kept dissolved in MetOH without undergoing a spontaneous gelation. Moreover, under these circumstances the average molecular weight of PHEMA chains is high enough so that they can be turned into a physically-intertwined network, if the solvent is partially or entirely removed. The resulting physical hydrogel is stable at physiological conditions—aqueous solutions and mild temperatures—and even irreversible when immersed in MetOH or DMF.

Even though this feature is a potential asset per se, such network deployability can be used for producing semi-interpenetrating networks of polymer with uneven side-chain hydrophilicity. That is the case of PHEMA and PCL, which can hardly be blended. Both have been widely studied in the biomaterials field, but their combinations addressed so far require complex reaction processes or in situ polymerisation [4,26,35], just the opposite of the current pursuit. PHEMA solutions with MetOH require a second predominant solvent (CHCl_3_) to embrace and dissolve PCL pellets, but once the quaternary solution is prepared, it can be easily spin coated to form substrates that are uniform beyond the customary criteria (nanophases of < 100 nm). Thus, the surface of the sIPN substrates presents a dual, amphipathic performance. Furthermore, PCL preserves their inherent ability to create crystalline segments in the sIPN, which in addition is presumably biodegradable because of the PCL chains and the potential reversibility of PHEMA networks.

sIPN interface with the milieu is not only amphiphilic, but also dynamic over time and dependent on the environment hydrophilicity, an appealing trait for interaction with proteins and their alternating sequences of aminoacids. For that very reason, not all compositions of PCL and PHEMA sIPN induced the same response in total mass of adsorbed proteins from bovine serum or the interfacial layout thereof. APsIPN80 substrates—i.e., those with 80% of PCL—proved to interact more markedly and steadily with the multiprotein medium, despite we found no evidences of clear imbalances between the relative compositions of the attached macromolecules. Inconsistencies on Figure 2a,b for PCL-rich samples are primarily attributed to differences between experimental protocols. That said, the possibility of this as a sign for different tertiary structures between adsorbed and denatured proteins should not be disregarded, after all, remaining specimens showed a strong correspondence. In fact, although all samples with PCL exhibited some sort of reticular protein structure after rinsing the serum with saline buffer, the surface onAPsIPN80 was the only one barely influenced by the deposition of proteins. The shape and distribution of this type of lattices is known to determine the functional domains revealed to the vicinities, the cue for cells’ fate when seeded on this type of substrates [36,37]. 

Electrospun meshes were successfully fabricated as well from APsIPN80 25% solutions as well, in CHCl_3_:MetOH 75:25 despite the apparent instability of the quaternary solutions and sharp shifts in rheological properties they undergo under subtle variations in composition, temperature or shear. This stimuli-responsiveness is the result of the close integration of the PCL and PHEMA moieties [33,35], but further studies should elucidate the optimal set of parameters more accurately and the impact of these meshes on cells when used as 3D culture platforms, to avoid unintended outcomes. Namely, high viscosities in the solution—just as electrospinning over 50 °C—involve a high risk of needle clogging, while a clear prevalence of the loss modulus (G”) is typically related to bead occurrence in fibres.

## Figures and Tables

**Table 1:** Effect of solvent fraction over resulting PHEMA polymerized at 60 °C for 24 h.

Solvent	Solvent Fraction ( wt %)							
	0	30	40	50	60	70	80	90
DMF	ISE	ISE	ISE	ISG	ISG	SG	SG	L
MetOH	ISE	ISE	ISE	ISG	ISG	SG	SG	L

ISE: Insoluble elastomer; ISG: Insoluble gel; SG: Soluble gel; L: Liquid.

**Table 2:** Effect of time over resulting PHEMA polymerized with 75 wt % solvent at 60 °C.

Solvent	Time (h)							
	0	4	8	12	16	20	24	28
DMF	L	L	L	L	SG	SG	SG	ISG
MetOH	L	L	L	SG	SG	SG	SG	ISG

ISE: Insoluble elastomer; NSG: Insoluble gel; SG: Soluble gel; L: Liquid.

**Figure A4:**
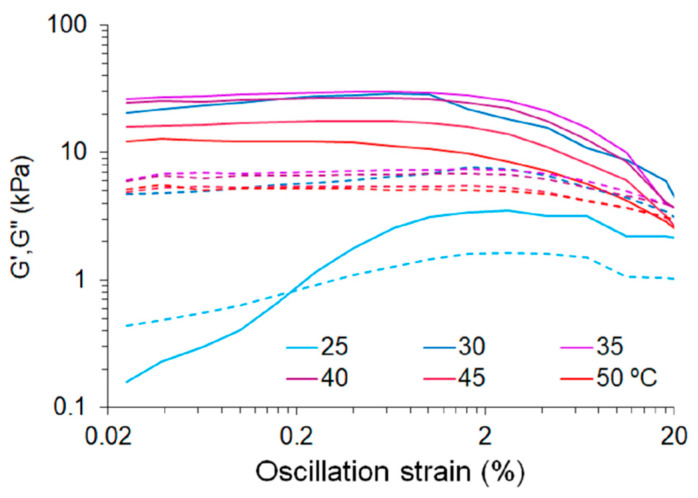
**Oscillatory rheology of APsIPN80 solutions.** Storage (G’, solid line) and loss (G”, dashed line) components of shear modulus versus oscillation strain for APsIPN80 12 wt % solutions in CHCl_3_:MetOH 3:1 at different temperatures.
